# The Tyrphostin Agent AG490 Prevents and Reverses Type 1 Diabetes in NOD Mice

**DOI:** 10.1371/journal.pone.0036079

**Published:** 2012-05-14

**Authors:** Abdoreza Davoodi-Semiromi, Clive H. Wasserfall, Chang Qing Xia, Rhonda M. Cooper-DeHoff, Martin Wabitsch, Michael Clare-Salzler, Mark Atkinson

**Affiliations:** 1 Department of Pharmacotherapy & Translational Research, College of Pharmacy, University of Florida, Gainesville, Florida, United States of America; 2 Department of Pathology, Immunology and Laboratory Medicine, University of Florida, Gainesville, Florida, United States of America; 3 Division of Pediatric Endocrinology, Diabetes and Obesity Unit, Department of Pediatrics and Adolescent Medicine, University of Ulm, Eythstr, Ulm, Germany; Children's Hospital Boston/Harvard Medical School, United States of America

## Abstract

**Background:**

Recent studies in the NOD (non-obese diabetic) mouse model of type 1 diabetes (T1D) support the notion that tyrosine kinase inhibitors have the potential for modulating disease development. However, the therapeutic effects of AG490 on the development of T1D are unknown.

**Materials and Methods:**

Female NOD mice were treated with AG490 (i.p, 1 mg/mouse) or DMSO starting at either 4 or 8 week of age, for five consecutive week, then once per week for 5 additional week. Analyses for the development and/or reversal of diabetes, insulitis, adoptive transfer, and other mechanistic studies were performed.

**Results:**

AG490 significantly inhibited the development of T1D (p = 0.02, p = 0.005; at two different time points). Monotherapy of newly diagnosed diabetic NOD mice with AG490 markedly resulted in disease remission in treated animals (n = 23) in comparision to the absolute inability (0%; 0/10, p = 0.003, Log-rank test) of DMSO and sustained eugluycemia was maintained for several months following drug withdrawal. Interestingly, adoptive transfer of splenocytes from AG490 treated NOD mice failed to transfer diabetes to recipient NOD.*Scid* mice. CD4 T-cells as well as bone marrow derived dendritic cells (BMDCs) from AG490 treated mice, showed higher expression of Foxp3 (p<0.004) and lower expression of co-stimulatory molecules, respectively. Screening of the mouse immune response gene arrary indicates that expression of costimulaotry molecule Ctla4 was upregulated in CD4+ T-cell in NOD mice treated with AG490, suggesting that AG490 is not a negative regulator of the immune system.

**Conclusion:**

The use of such agents, given their extensive safety profiles, provides a strong foundation for their translation to humans with or at increased risk for the disease.

## Introduction

Tyrosine phosphorylation inhibitors, termed tyrphostins, are a family of low molecular weight molecules having the ability to inhibit tyrosine phosphorylation through protein tyrosine kinases (PTKs). The family has at least 31 members, with AG490, also known as tyrphostin B42, being one [1]. AG490 has been widely studied in cancer and autoimmune diseases, but not subject to investigation in the setting of T1D. Tyrosine kinase inhibitors (TKIs) have been broadly used in many cancer clinical trials [2]–[4], T1D [5], [6] in mouse model of multiple sclerosis [7] and in intestinal inflmmation is reported [8]. Oral administration of Sunitinib (Sutent) and Imatinib (Gleevec), two TKIs, have been shown to prevent and reverse T1D in the NOD mouse [5]. In several publications successful remission of T1D has been reported [9]–[16]; however, when it comes to monotherapy approach long-lasting remission of diabetes is still challlenging [17].

The Jak-Stat signaling pathway plays a critical role in mediating inflammatory immune response to a variety of signals [18]–[21]. Currently, several Jak-Stat inhibitors have been developed and are under clinical investigation [4], [22]–[25]. AG490 blocks the activation of the Jak and STAT family of molecules [1]. AG490 has been reported to be more potent as a Jak2 inhibitor (4.3-fold) than Jak3 [26]; however, it has no impact on membrane associated kinases such as Lyn, Btk, Syk, Src and Zap70 [27], [28] suggesting that it is not a general kinase inhibitor [26], [27], [29]–[31]. Additionally, AG490 blocks tumor growth and inhibits/delays the onset of autoimmune diseases such as experimental autoimmune encephalomyelitis (EAE) [32], inhibits antigen-specific T-cell infiltration in EAE mice [32]–[34], and significantly extends rat heart allograft survival [35]. AG490 also blocks IL-12 mediated T cell proliferation, inhibits phosphorylation of Stat3, decreases IFN-γ production mediated by IL-12 [32], and inhibits differentiation of antigen-specific Th1 cells *in vivo* [36]- all suggesting that this drug possess immunomodulating properties.

Previously, we reported several defects in the Jak-Stat5 signaling pathway in NOD mice including autoactivation of Stat5, germ-line mutation in Stat5B and impaired DNA binding of Stat5B in NOD mice [37]–[39]. Given the influence of AG490 on the Jak-Stat pathway, we sought to block the Jak-Stat signaling pathway and investigate the effects of this compound on early (4weeks) and late model of T1D development (8 weeks), insulitis and initiation of remission of newly diagnosed and established diabetic NOD mice.

## Results

### AG490 prevents development of autoimmune diabetes in NOD

**Figure 1:**
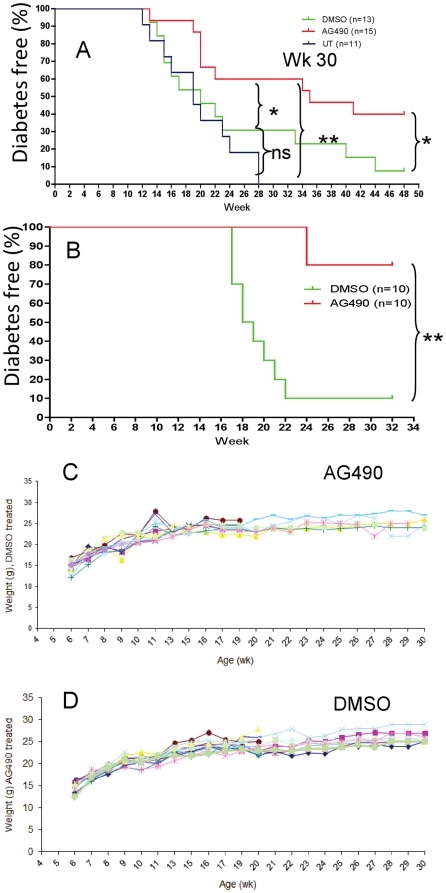
AG490 prevents development of autoimmune diabetes. Prediabetic female NOD mice at 4 (UT: n = 11; DMSO: n = 13; and AG490 treated mice: n = 15 mice/group) (A), or at 8 week of age, (n = 10 per group) (B) were treated with AG490 or DMSO (i.p) for 5 consecutive week, three time per week followed by once a week for 5 more week [Kaplan-Meier test (*p = 0.02, 1A) and (**p = 0.005, 1B, at week 30)]. Our data also indicate that significant differences (p<0.01) was observed between the AG490 and DMSO treated mice for up week 52; when treatment was started at week 4. Body weight of NOD mice treated with AG490 (C) or DMSO (D) in mice when treatment initiated from 4 weeks of age is shown. [This figure is intended to be in color online and black and white in print.]

Prediabetic female NOD mice were treated with AG490 (i.p, 1 mg/mouse) and control mice received the same volume of vehicle (DMSO, solvent of AG490, and sterile PBS) three times/week for 5 consecutive week and once a week for five more week starting at 4 (preinsulitis) or 8 week of age (established insulitis). Our data suggests that AG490 prevents development of autoimmune diabetes in NOD mice when applied at 4 or 8 week of age (p = 0.02 and p = 0.005, respectively at week 30) (figure 1A & B). Blood glucose and body weights (figure 1C and D) were monitored in both groups as early as 6 week of age and continued for the entire period of investigation. There was no statistically significant differences in terms of body weights between AG490 and DMSO treated mice suggesting administration of AG490 at the given dose is safe in mice. Additionally, we also did not observe any adverse effect of AG490 treated mice by gross examination suggesting that efficacy and safety of multiple injections of AG490. This data suggests that AG490 is an efficient and safe drug in prevention of diabetes in NOD mice.

### AG490 induces remission of autoimmune diabetes in NOD mice

**Figure 2:**
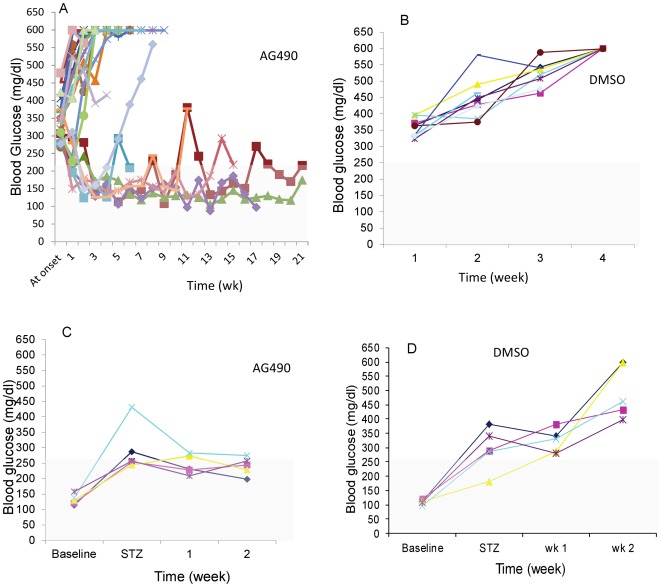
AG490 induces remission of diabetes in NOD and in STZ-induced diabetic mice. Newly diagnosed and established diabetic NOD mice were treated with AG490 (i.p, 1 mg/mouse) without insulin supplement (A) (n = 23) or with DMSO (B) (n = 10). Blood glucose was measured by ACCU-CHECK 2–3 times weekly. Sustainable euglycemia was observed for several months after drug withdrawal while DMSO treated mice expired within 2–4 week after disease onset. The average blood glucose at the disease onset in the cured NOD mice was 314.43 mg/d (Max = 374; Min = 277 mg/dl) versus 384.58 mg/dl (Max = 478, Min = 335 mg/dl) in mice that AG490 failed to restore euglycemia (p<0.0014). An average blood glucose in cured NOD mice treated with AG490 was 169.82 mg/dl±23.21, n = 7 versus 555.13±63.79, n = 17 in uncured NOD mice (p = 1.15E-11). (C) Diabetes was induced by STZ injection in male BALB/c mice and one week after the last injection diabetic mice were treated with either AG490 or DMSO (D) (n = 5/group). The blood glucose level was significantly lower (p<0.004) in AG490 treated mice when compared with DMSO treated mice. [This figure is intended to be in color online and black and white in print.]

We sought to investigate whether AG490 would reverse pathogenesis of T1D in newly diagnosed diabetic NOD mice. Newly dignosed and established diabetic NOD mice were treated with AG490 (i.p, 1 mg/mouse; no insulin was given to mice at any time) and control group was treated with vehicle and blood glucose was measured. Our data indicate that AG490 led significantly to remission of diabetic mice (p = 0.003, Log-rank test) while DMSO treated control mice led to remission of 0% (n = 10) (figure 2A & B). Once euglycemia was established (Table S1), treatment was ceased and normaglycemia lasts for several weeks and sustainable euglycemia was maintained after drug withdrawal in majority of the cured mice. These data suggest that NOD mice did not develop resistance to AG490 treatment and additionally suggests that AG490 has a durable effect on restoration of blood glucose metabolism/regulation. However, diabetic NOD mice treated with DMSO or left untreated expired within 2–4 weeks (figure 2B). Average of blood glucose in the cured NOD mice at the disease onset was 314.43 mg/dl (Max = 374; Min = 277 mg/dl) versus 384.58 mg/dl (Max = 478, Min = 335 mg/dl) (p<0.0014) in mice that AG490 failed to restore euglycemia while an average blood glucose in cure NOD mice was 169.82±23.21 mg/dl versus 555.13±63.79 (p = 1.15E-11) in uncured NOD mice. This data suggest that AG490 was more effective in diabetic mice with blood glucose level <314.43 mg/dl. A combination therapy, e.g AG490+ insulin, may improve efficacy of AG490 treatment in mice with blood glucose level >314.43 mg/dl.

### AG490 treatment did not cause clinical signs of toxicity and behavior

Treatment-related mortality in the course of i.p (intraperitoneal) injection of AG490 and thereafter did not observe. Clinical signs of toxicity related to treatment such as loss of body weight, strange behavior such as aggressiveness, hypermobility, hunchback, and other signs such as discoloration of the fur or fur loss, urine and stool discoloration did not observed.Treatment did not change apetite and food/water consumption as judged by body weight gain/loss as measured 1–2 times per week and continued throughtout the study and no differences between AG490 and DMSO treated mice were observed (see figure 1C and D) owning to the good general condition of animls. Laboratory examination such as hematology, clinical chemistry and urine analysis did not perform.

### AG490 induces remission of diabetes in STZ induced diabetic mice

To futher explore whether AG490 can reverse diabetes in STZ-induced diabetic mice, BLAB/c mice were treated with STZ and then were treated one week ater the last STZ injection either with AG490 or DMSO (n = 5/group). Our data indicate that the mean blood glucose in DMSO treated mice was significantly higher than AG490 treated mice after 2-weeks of treatment (DMSO: 498.6±29.3 mg/dl; AG490: 240.4±95.2 mg/dl; p<0.004) (figure 2C & D). This data further suggests that AG490 is an effective and safe drug to reverse onset of diabetes and further is suggesting that the Jak-Stat signaling pathway may have additional role(s) in pathogenesis of diabetes and on glucose metabolism than currently appreciated.

### Adoptive transfer of AG490 treated splenocytes did not transfer diabetes in NOD.*Scid* mice

**Figure 3:**
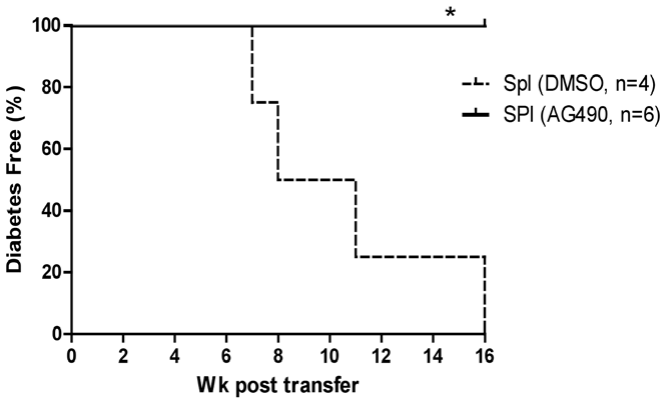
*In vivo* AG490 treated splenocytes did not transfer diabetes to NOD.*Scid* mice. Prediabetic NOD mice (4- weeks of age) were treated with AG490 or DMSO for five consecutive week (n = 5 per group), three times per week and mice were sacrificed one week after the last injection at week 10. Splenocyte cell suspension of pooled mice immediately prepared and dead cells and red blood cells were removed by Histopacque gradient separation method and freshly isolated splenocytes containing >99% viable cells were transferred (5×10^6^, i.p) into NOD.*Scid* mice. Blood glucose in recipient mice was monitored at least once a week as described earlier (*: p = 0.02, Kaplan-Meier test).

In order to understand how AG490 reversed and blocked the onset of autoimmune diabetes *in vivo*, we treated prediabetic NOD mice (4-week of age) with AG490 or with DMSO for 5 week. Mice were sacrificied at week 10 and splenocyte cell suspensions were prepared with Histopaque gradient separation. The dead cells and reticulocytes were removed by the Histopaque gradient separation, therefore, dead cells were excluded without magnetic purification and without activation of T-cells and cell viability was greater than 99%. Five million freshely prepared of pooled splenocytes of NOD mice treated with AG490 (n = 5) or DMSO (n = 5) were transferred by i.p injection into recipient NOD.*Scid* mice and blood glucose and body weight was closely monitored. Our data as shown in figure 3 suggests that prevention of autoimmune diabetes by AG490 could be due to inactivation of atuoreactive T-cells and/or induction/generation of regulatory T-cell or other regulatory population.

### Administration of AG490 does not diminish leukocyte infiltration within the pancreas

**Figure 4:**
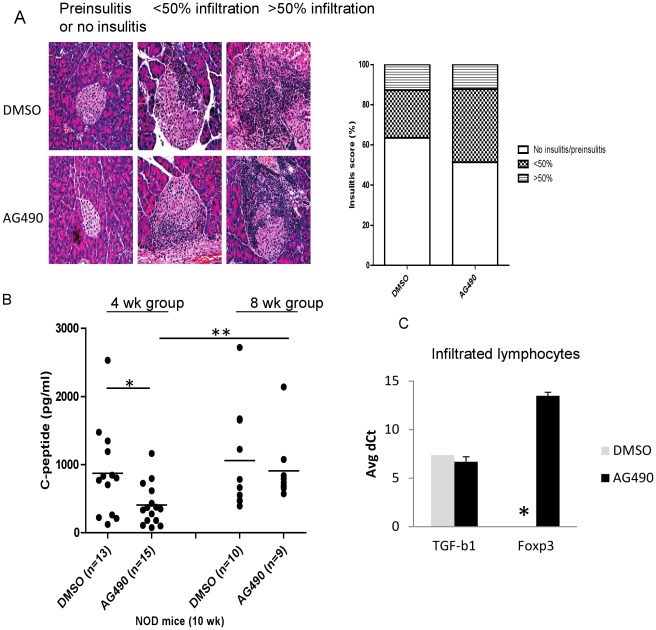
AG490 does not block infiltration of leukocytes in the islets. A- Prediabetic NOD mice at 4 weeks of age were treated with AG490 or DMSO (n = 5 per group) for 5 consecutive weeks (3× per week) and mice were sacrificed one week after the last injection at week 10. Insulitis was scored by two different investigators and an average of two different scores is shown (p = n.s). At least 150 islets per group in two interrupted sections, 100 µm apart, was scored. B- The C-peptide level was measured as described in materials and methods and no significant differences were found. C- Five prediabetic NOD mice at 4- weeks of age were treated with AG490 or DMSO (n = 5 per group) as described above for 5 consecutive weeks and then sacrificed at week 10, C- real-time RT-PCR was performed on enriched infiltrated lymphocyte from the pancreas of 5 mice/group and expression level of Foxp3 and TGF-β1 was measured in three independent RT-PCR by TaqMan Gene expression (Life Technologies, Applied Biosystems, Assays-on-Demand) (* = expression was very low and/or undetectable). [Figure 4A is intended to be in color online and black and white in print.]

In another separate experiment, NOD mice at 4 week of age were treated with AG490 or with DMSO (n = 5/gorup), three times per week for 5 consecutive week. Mice were sacrificed at week 10 and microscopic sections were prepared and stained with H & E and scoring of insulitis was performed as described in materials and methods (figure 4A). There was no statistically significance differences regarding insulitis between the two groups. The C-peptide concentration was significantly different in AG490 treated versus DMSO treated mice (4 week group); however, no significant differences in serum C-peptide concentration of AG490 or DMSO treated NOD mice (8 week group) was found (figure 4B). The C-peptide concentration of AG490 treated NOD (8 week group) was significatly higher than the C-peptide level of mice when treatment was initiated at 4 weeks of age. In a separate experiment, female NOD mice were treated either with AG490 or DMSO for 5 weeks and then were sacrificied at week 10. Total RNA extracted from enriched infiltrating leukocytes from pancrease of AG490 and DMSO treated mice (n = 5 mice/group) was subjected to TaqMan Real-time RT-PCR. Level of Foxp3 and TGF-β1 expression were very low but detectable in AG940 treated NOD mice but we failed to detect expression of Foxp3 in three independent RT-PCR in DMSO treated NOD mice (figure 4C). This data suggests that although administration of AG490 was unable to block infiltration of the leukocytes in the islets or improve the C-peptide concentration; however, it changed composition of the infiltrated leukocytes in the islets.

### Administration of AG490 does not block expression of Jak-1 and Stat-1 in leukocytes infiltrating the islets

Expression of the Jak and Stat family members in the islets of NOD mice treated with AG490 or DMSO were screened by immunofluorescen histochemistry technique. Expression of Jak3, Stat3 and Stat5 was very low/undetectable in AG490 treated mice. Expression of Jak2 was undetectable or it was very low even in DMSO treated NOD mice. AG490 had no effect on constitutive expression of the Jak1 and Stat1(figure S1). Higher expression of Jak1 and Stat1 is of importance because it is known that IFNγ uses JAK-1 for activation of Stat1 [19], [21], [40]. Additionally, since adminsitration of AG490 might inhibit expression of other kinases than members of the JAK family, we screened expression of EGFR, c-Kit, PDGFR and c-Able by western blot in splenocytes of NOD mice treated with AG490 or DMSO (n = 5). In several independent western blots, all detected bands did not match with expected molecular weights of those protein suggesting that expression of these kinases was very low and AG490 likely does not have any impact on expression of these kinases.

### Administration of AG490 significantly increases expression of Foxp3

**Figure 5:**
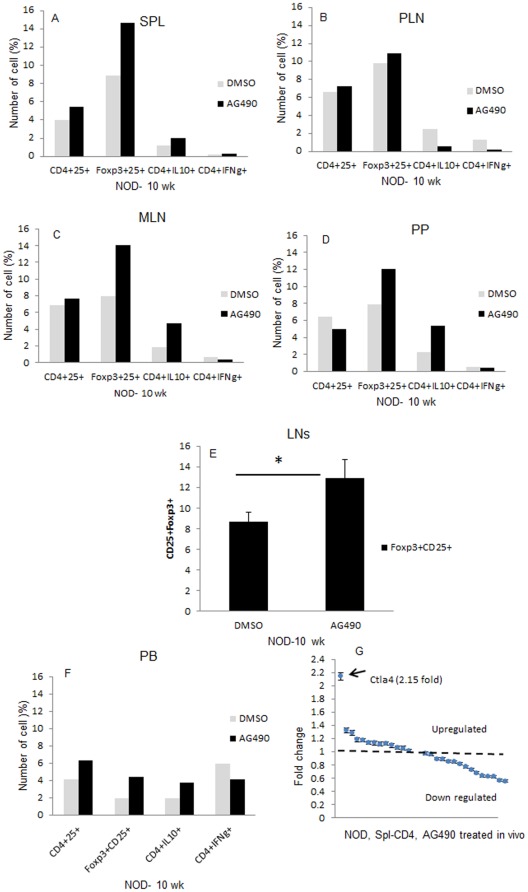
AG490 upregulates expression of Foxp3 regulatory CD25+ T-cells *in vivo*. A- Prediabetic NOD mice (4 week) were treated either with AG490 or DMSO three times per week for 5 consecutive weeks and they were sacrificed one week after the last injection at week 10. Data represents pool cell suspensions of 5 NOD mice per group and is gated on live CD4+ T-cells. AG490 treatment significantly increases number of Foxp3 regulatory T-cells (p<0.004) (A–D). E- CD25+Foxp3+ gated population on CD4+ T-cell of several lymph nodes in AG490 and the sham treated control NOD mice. Bars represent standard deviation of the mean. F- Whole blood of NOD mouse (pool of 5 mice/group) treated as described above was stained directly with antibodies indicated as described in materials and methods. Data of gated CD4+ T-cells are shown. G- Total RNA from purified CD4+ T-cells of splenocytes of NOD mice (10 weeks old) treated either with AG490 or DMSO from 5 weeks were subjected to real-time RT-PCR and expression of the immune response genes was screened as described in materials and methods. Data shown is the mean of fold change expression of 18 genes after they were normalized based on expression of two housekeeping genes and then compared with the expression of the same gene in DMSO treated control NOD mice. Spl = spleen, PLN = pancreatic lymph node, MLN = mesenteric lymph node, PP = Peyer's patches, PB = peripheral blood.

To further understand as to whether AG490 prevented and reversed diabetes in treated NOD mice, we measured expression of Foxp3 and other regulatory markers by flow cytometry analysis in different organs of the immune system in NOD mice that were treated with either AG490 or DMSO. As shown in figure 5A–D significant higher expression of CD25+Foxp3+ T-cells (p<0.004) is evident in all organs tested in AG490 treated mice. Furthermore, AG490 treatment also increased expression of Foxp3 in CD25+ T-cells in peripheral blood of the same groups of NOD (Fig. 5E).

### AG490 is not a negative regulator of the immune system; screening of the mouse immune response genes array

In a separate experiment, pooled total RNA extracted from purified CD4+ T-cells from spleen of the AG490 or DMSO treated NOD mice (treated 3x/week for 5 weeks and euthanized at week 10, n = 5/group) were used to screen the mouse immune response genes array (Applied Biosystems) by real-time TaqMan Gene Expression RT-PCR. As shown in figure 5F, AG490 did not negatively regulate overall gene expression of the mouse immune response genes (see Table S2). Interestingly, *in vivo* treatment of NOD mice with AG490 increased (2.15 fold) expression of co-stimulatory *Ctla4* in CD4+ T-cell. The *Ctla4* gene is a diabetes candidate gene for T1D and its association is well documented in the literature [41]–[44]. Global gene expression screening from different cellular component of the immune system of NOD mice treated with AG490 is warranted to better understand mechanism underlying modulatory function of AG490 and that may lead to identify its target gene(s)/pathway(s).

### *In vivo* and *in vitro* administration of AG490 modulates phenotype and function of dendritic cells

**Figure 6:**
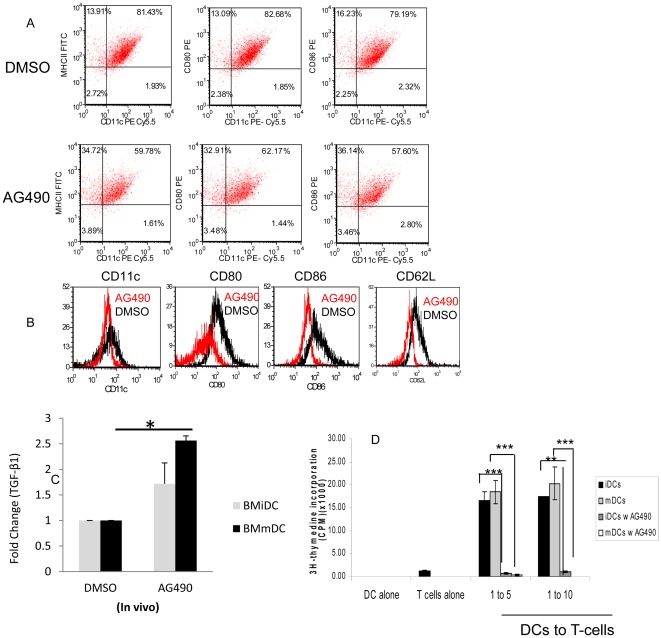
AG490 modulates phenotype and function of DC. A - Dendritic cells were generated from bone marrow of NOD mice treated either with AG490 or DMSO as described in materials and methods and stained directly with CD11c, MHC II, CD80 and CD86. Data shown represents CD11c gated population of pool fresh purified immature CD11c of AG490 and DMSO treated mice. B- Histograms represent expression of CD11c, CD86, CD80 and CD62L on gated CD11c population of mice treated with AG490 or DMSO *in vivo*. C- Bone marrow cells were isolated from NOD mice treated with AG490/DMSO for 5 weeks and then differentiated into DC *in vitro* as described. Total RNA (200 ng) of DC was converted into cDNA and then expression of Tgf-β1 was measured by Real-Time TaqMan Gene expression assays (Applied Biosystems). Data represents the mean (fold change) of two real-time RT-PCR experiments and the bars represent standard deviation of the mean. Fold change of Tgf-β1expression is shown (n = 5 mice/group, * p = 0.02). D- BMDCs were generated from BALB/c mice treated *in vitro* with 20 µM AG490 or DMSO for 12 hrs and then co-cultured with constant numbers of CD4+CD25− T-cells (1×10^5^) with soluble anti-CD3 (2.5 ng/ml) for 72 hrs. Incorporation of ^3^H-thymidine was measured in the last 16 hr of cell culture. Experiment was performed in triplicate format and repeated at least twice. Data analysis was performed using the student *t*-test and p value≤0.05 considered significant. Bars represent deviation of the mean (* p = 0.002, **p = 0.0002; ***p = 0.001). [Figures A & B are intended to be in color online and black and white in print].

Bone marrow of AG490 and DMSO treated NOD mice (4 weeks/3x/week, treated for 5 weeks) were harvested at week 10 and yeilded the same number of cells in each group (*p* = not significant). We did not, however, notice any remarkable difference in morphology, such as the size, shape and clonoy formation, between the two groups in the course of differentiation in cell culture. As shown in figure 6A and B, BMDCs (bone marrow derived dendritic cells)of mice treated by AG490 had markdly lower expression of co-stimualtory moelcules CD80 and CD86 and adhesion molecule CD62L. Additionally, our real-time TaqMan Gene expression Asssays (Assays-on-Demand) (Life technologies, Applied Biosystems) indicates that AG490 significantly increased expression of regulatory cytokine Tgf-β1 in mature and immature DC (dendritic cell) when it was compared with the sham treated control NOD mice (figure 6C, n = 5/group, p = 0.02). Furthermore, BMDCs treated with AG490 (overnight, 20 µM) or DMSO suppressed proliferation of purified responding CD4+CD25− T-cells *in vitro* (figure 6D).

## Discussion

Presence of proinflammatory cytokines due to ongoing inflammation in NOD mice, leads to autoactivation of different members of the Jak and Stat family followed by activation of downstream events as it has been reported by us [37], [45] and others [46]. Recently, it has been shown that a transcriptomic analysis of glomerular and tubulointerstitial tissues from diabetic subjects with early and progressive diabetic nephropathy revealed involvement of multiple members of the Jak/Stat signaling pathway [47]. Interestingly, higher expression of the *Stat5B* in progressive diabetic nephropathy patients has been shown [47].

Tyrosine kinase inhibitors have been reported to decrease insulin resistance [48] and increase insulin secretion *in vitro* [49], [50]. In our study, AG490 decreased hyperglycemia in chemically induced diabetic mice and in naturally developed diabetic NOD mice. In chemically induced study, since the treatment with AG490 was started one week after diabetes onset, beta-cell destruction was more or less completed at this stage; therefore, improvement in blood glucose level is the result of increased insulin sensitivity by AG490. Rapid recovery from diabetes in response to AG490 therapy, inefficiency of AG490 on the C-peptide levels, the lack of any AG490 effect on leukocyte islet infiltration- are all in agreement with improved insulin sensitivity in peripheral target tissues. On the other hand, Real-time RT-PCR data consistently indicates that expression of Foxp3 in infiltrating lymphocytes in the pancreas of AG490 treated NOD mice was detectable while it was undetectable in DMSO treated mice (see figure 4C). This data suggests that AG490 treatment changed composition of infiltrating leukocyte of the islet in NOD mice.

Our previous reports regarding autoactivation and impaired function of the Jak-Stat pathway [37], [45] and our data in this study-all may suggest that the Jak-Stat signaling pathway may be an excellent target for immunotherapy of T1D and may also be effective in type II diabetes, although this remains to be seen [51]. Autoactivation of kinases/transcription factors due to ongoing autoimmunity will generate inflammatory cytokines which subsequently will lead to further inflammation. The Jak-Stat signaling pathway is the most important signaling pathway for transduction of signals from the cell surface to the nucleus [21], [52]–[54]. This signaling pathway is involved in the generation of pro-inflammatory cytokines and is bound to have many more important roles in the pathogenesis of T1D than currently is appreciated. Our study uncovered several novel features of AG490 such as prevention of diabetes, initiation of remission in newly diagnosed NOD mice and in pharmacological induced diabetic mice, increasing number of Foxp3 regulatory T-cells and probably generation of regulatory DC. It is important to mention that significant differences (p<0.01) in incidence of diabetes was observed between the AG490 and DMSO treated mice for up to 52 week when treatment was started at week 4. This data may suggest that appropriate blocking of the Jak-Stat signaling pathway could bring about a durable and long-lasting tolerance and further is supporting the notion of safety and efficacy of AG490 in prevention of autoimmune diabetes in NOD mouse.

Our data suggest that administration of AG490 blocks expression of several members of the Jak and Stat families in infiltrated leukocytes in the islets of NOD mice. Lack of the Stat3 expression is fatal perinataly [55], however, none of the treated mice in this study died because of AG490 treatment suggesting that treatment by AG490 was not life threatening. Our microarray real-time TaqMan gene expression data suggests that AG490 may exert its regulatory effect via upregulation of negative co-stimulatory molecule, *Ctla4*, in CD4+ T-cells; however, additional screening of different cellular component of the immune system is warranted to identify it target gene(s)/pathway(s) and better understand its molecular mechanism. Our data indicate that body mass in AG490 and the sham treated control mice was not significantly different from each other suggesting that AG490 treatment did not cause obesity [56].

In this study, we demonstrate that AG490 initiates remission of diabetes in newly diagnosed and established diabetic mice, no insulin was injected at any time, and sustained euglycemia was evident for several months following drug withdrawal. It has been shown that AG490 increased glucose uptake (102±9) in L6 myotubes [48] under high glucose concentration in comparison with vehicle-treated cells [57]. AG490 decreased fibronectin expression; higher expression of fibronectin is associated with nephropathy in diabetic subjects, under high glucose condition in glomerular mesangial cell; therefore, AG490 potentially could be an effective treatment for prevention of diabetic nephropathy [58]. Adoptive transfer of splenocytes isolated from in vivo treated mice with AG490 failed to transfer diabetes to immunodeficient NOD mice supporting the notion of inactivation of autoreactive T-cell or induction of regulatory T-cell by AG490. DC plays a central role in induction and maintenance of the central and peripheral tolerance [59], [60] and its development is impaired in NOD mice [61]. As shown in this study, DC treated with AG490 possesses regulatory function and suppress proliferation of responding T-cells *in vitro*. Recently, Louvet et al have reported successful reversal of diabetes by oral administration of tyrosine kinase inhibitors [5], however, diabetes was transferred from drug and the sham treated control mice into immunedeficient hosts with the same kinetics [5] suggesting that induction of tolerance was achieved by other means than generation of the regulatory T-cells.

In summary, this study provides evidence regarding application of AG490 in prevention/reversal of autoimmune T1D in NOD mouse. Administration of AG490 *in vivo* was very well tolerated and treated NOD mice with AG490 showed no signs of toxicity and they appeared healthier as they had a longer life span and they were significantly diabetes-free when compared to the sham treated or untouched control NOD mice. Additionally, this study provides evidence regarding the crucial role of the Jak-Stat signaling pathway in development of autoimmune diabetes and regulation of blood glucose. Therefore, the Jak-Stat signaling pathway may be an excellent pathway to be targeted for immunotherapy and/or to reverse diabetes. The use of such compounds like AG490, given their extensive safety profiles, offers a solid foundation for their translation to diabetic subjects with or at increased risk for the disease.

## Materials and Methods

### Ethics statement

All mice were maintained and handled in accordance with the University of Florida Institutional Animal Care and Use Committee (IACUC), who specifically approved this study.

### Mice

Female NOD/LtJ, NOD.*Scid*, and BALB/c mice were purchased from Jackson Laboratories (Bar Harbor, ME) and housed in ventilated cages in a specific pathogen free facility.

### Compound

AG490 (EMD Chemicals, Gibbstown, NJ), 5 mg, initially was dissolved in sterile DMSO (EMD Chemicals) and brought to final volume by sterile PBS followed by several pipetting to bring the compound into solution. One vial of compound containing 5 mg of AG490 was injected into5 mice (1 mg/mouse) via the i.p route. The control groups were received the same volume of the vehicle under the same regimens and conditions.

### Administration of AG490

Mice were injected either at 4 or 8 weeks of age, three times per week for 5 consecutive weeks followed by 5 more injections, once a week for 5 additional weeks. Body mass and blood glucose levels were measured at the beginning of the study (baseline) and monitored at least twice a week starting at week 6 and continued thereafter. Blood glucose concentrations were determined using ACCU-CHEK Comfort Curve (Roche, Indianapolis), and diabetes diagnosed if values were greater than 250 mg/dl in two consecutive tests with 24 hours apart.

### Tolerability and clinical signs of toxicity

Clinical signs of toxicity related to treatment such as weight gain/loss, abnormal behavior, discoloration of urine, stool and fur monitored at least 3 times per week and continued throughout the study. Body weight was recorded before initiation of the study and measured 1–2 times weekly thereafter.

### Induction of diabetes by i.p injection of streptozotocin (STZ)

STZ (Sigma-Aldrich, St. Louis, MO) was freshly prepared in citrate buffer (pH = 4.5) just before injection and final volume was adjusted by adding sterile PBS. BALB/c mice (n = 10) were received 5 dose of STZ (50 mg/kg) by i.p injection in 5-consecitive days. Diabetic mice were treated one week post diabetes onset with either AG490 or DMSO (control).

### Purification of CD4+, CD4+CD25+/− T-cells and dendritic cells

Purification of CD4+CD25+ T-cells was performed from pooled splenocytes using a CD4+CD25+ isolation kit (Miltenyi Biotec, Auburn, AL) and autoMACS magnetic separator, according to instructions provided by the manufacturer. Purity of purified CD4+CD25+ and CD4+CD25− T-cells were greater than 85–90% and 95%, respectively, as judged by flow cytometry analysis. Splenic CD4+ T-cells and dendritic cells (from spleen and bone marrow derived DCs) were enriched with CD4+ and CD11c MicroBeads, respectively (Miltenyi Biotech) using autoMACS instrument according to manufacturer's instructions.

### Generation of bone marrow-derived dendritic cells

Dendritic cells (DC) were generated from bone marrow in 5days. In brief, mice were euthanized and femur and tibiae removed and cleaned from other tissues. Both ends of each bone were cut and marrow flushed with RPMI 1640 containing 10% serum. Red blood cells were lysed with an ammonium chloride lysis solution (Sigma-Aldrich), and cells were plated at 1×10^6^ cells/ml, 2 ml/well in 12-well plate (Falcon; BD Biosciences) in RPMI 1640 medium, supplemented with 1% penicillin and streptomycin, 10% heat-inactivated and filtered FCS containing rmGM-CSF (500 U/ml R&D systems) and IL-4 (1000 U/ml, BD Biosciences). On day 2, non-adherent and loosely adherent cells were harvested by replacing one-half of the cell-containing supernatant from each well and replaced with 1 ml of fresh medium containing cytokines. On day 5, DC maturation proceeded by adding of LPS and those cells that did not receive LPS were considered as immature DC. Daily microscopic inspection of cell culture by microscopic examination was performed until the end of the DC culture.

### T- cell culture, Proliferation/Suppression assays

The AG490/DMSO treated bone marrow derived DC (BMDC) were cultured at different ratios, with constant number of CD4+CD25− T-cells (1×10^5^), in 96-well round bottom plates containing soluble anti-CD3 (2.5 µg/ml) for 72 hourrs. Proliferation of responding populations were measured in the last 16 hours of cell culture by measuring ^3^H-thymidine incorporation into the DNA using Scintillation Counter (Beckman).

### TaqMan mouse Immune Response Array and Real-Time TaqMan Gene expression analyses

Total RNA was extracted from CD4 and dendritic cells using RNAeasy kit (QIAGEN). CD4+ T-cells was purified from 5 individual mice using mouse CD4+ T-cell isolation kit (Miltenyi Biotech) followed by autoMACS separation according to the manufacturer's instructions. To screen the Taqman Array Mouse Immune Response, 200 ng of total RNA was converted to cDNA using High Capacity cDNA Reverse Transcription kit (Applied Biosystems). The cDNA from 5 mice were pooled together (one microgram of total RNA) and then used to screen 96- immune response related genes including 4 housekeeping genes. In the case of Real-Time TaqMan Gene expression (Assay-On-Demand), 100 ng of total RNA was used for reverse transcription followed by PCR amplification using 7300 Applied Biosystems PCR machine. All primers and probes were purchased from Applied Biosystems (Assays-on-Demand).

### Adoptive transfer

Female NOD mice (4 weeks old) were treated with AG490 or DMSO (n = 5 per group) for 5 consecutive week, with three injections per week. Mice were sacrificed one week after the last injection (i.e., week 10). Cell suspensions of splenocytes from pool mice were prepared, with dead cells and red blood cells removed by Histopaque (Sigma) gradient separation. Cell viability was >99% as judged by trypan blue exclusion. 5×10^6^ splenocytes were transferred by i.p injection into female NOD.*Scid* recipient mice.

### Flow cytometry analysis

Cell surface staining was performed using anti-mouse CD4 (L3T4; BD Pharmingen, San Diego, CA), CD25 (3C7; BD Pharmingen), CD11c (HL3; BD Pharmingen), CD80 (16-10A1; eBioscience, San Diego, CA), biotin-conjugated MHC II (M5/114.15.2; eBioscience) and anti-hTGFβ- Biotin (BAF240; R & D Systems) and biotin labeled anti chicken IgY (BAF010; R & D System) for isotype control. Purified rat anti-mouse CD16/CD32 (2.4G2; BD Pharmingen) was used to block Fc receptor in myeloid cell lineages. Intra-cellular staining of Foxp3 (FJK, 16S), IL-10 (JES5-16E3), IFNγ (XMG1.2) was performed using Foxp3 intracellular staining kit (eBioscience), according to instructions provided by the manufacturer. Viable cells were gated and a minimum of 30,000 events were acquired using a FACScaliber (BD Biosciences). Data was analyzed using FCS Express (v3) software (De Novo Software, Los Angeles CA).

### Histology and immunofluorescence histochemistry (IFHC)

Pancreata were fixed in 4% PFA (paraformaldehyde) and microscopic sections prepared with 100 µm interval from paraffin embedded tissue, and stained with H & E, at the Pathology Core Laboratories at University of Florida. Insulitis scoring was performed in a blinded fashion. A numeric code was given to each slide and then scored by two different investigators. A three point scoring method was used where 0 was given to pre-insulitis or intact islets, 1 was given to islets with <50% infiltration and 2 where infiltration was >50%. IFHC was performed on 4 µm paraffin sections of pancreas from the NOD mice. The concentration of primary antibodies was based on recommendation of the manufacturers. Stat1p91 (SC-591), Stat3 (SC-7179), Stat5B (SC-835), IFNγ (SC-8308), insulin (SC-7838), Jak1 (SC-277), JAk2 (SC-1656), Jak3 (SC-6932), Tyk2 (SC-136265), were from Santa Cruz (Santa Cruz, CA) and were diluted 1∶ 50 and TNFa-PE conjugated Ab (Pharmingen) was diluted 1∶100. Secondary fluorescence conjugated antibodies with Alexa Fluor 488 (green) (1∶ 200) or Alexa Fluor 594 (red) (1∶ 500) (Invitrogen, Carlsbad, CA) against the host primary antibody were used for signal detection. Anti-fade solution (Invitrogen) containing DAPI was used for counter staining and mounting.

*Western Blot-* Total protein was extracted from spleen of NOD mice treated either with DMSO or AG490 using RIPA lysis and extraction buffer containing Halt Protease and phosphatase inhibitor cocktail (Thermo Scientific). The following primary antibodies were used in this study: anti- mouse CD140b (PDGFRb), anti- mouse CD117 (c-kit) from eBioscience, and anti-mouse Abe and EGFR (BD Bioscience), anti-phosphoPPARγ antibody (Millipore, Billerica), anti PPARγ antibody (R & D Systems), and HRP conjugated antibodies (Sigma) with ECL detection kit (Millipore) were used for signal detection.

### Statistical analysis

All analyses for statistical significance were performed using Prism 5.0 software (GraphPad Software Inc, La Jolla, CA). The *t*- tests used for comparison of the mean for two groups and statistical comparison among different groups were performed using one-way analysis of variance (ANOVA) with Tukey's and/or Dunnett's post correction test for multiple comparisons, and/or for comparison with a control group, respectively. The Real-Time data (RQ values, fold change) was calculated using integrated SDS software, V1.4.0 (Applied Biosystems) and as described elsewhere [62]. The ΔC_t_ for each treated samples was compared with the mean ΔC_t_ of control samples (unstimulated) using the relative quantitation 2^−(ΔΔCt)^ method as described elsewhere [62]. *In vivo* data was analyzed using Kaplan-Meier test. A *P* value less than 0.05 was considered significant.

## Supporting Information

Figure S1 **The effects of AG490 on members of the Jak-Stat in the pancreas.** Prediabetic NOD mice (4 week) were treated either with AG490 or DMSO three times per week for 5 consecutive weeks and were sacrificed one week after the last injection at week 10. Pancreata were fixed and immunofluorescence staining was performed on microscopic slides as described in materials and methods. At least 30 islets per marker per group were analyzed.
(DOCX)

Table S1 **AG490 reverses diabetes in NOD mice.** Reversal of diabetes was performed by administration of AG490 via i.p route. Recently diagnosed diabetic NOD mice (n = 23) were under AG490 treatment for a period of 21 weeks. The age of disease onset, blood glucose level at disease onset and the mean of blood glucose level after treatment and also total number of AG490 injections are shown. Bold cases represent NOD mouse in which AG490 was effective and successfully established euglycemia (n = 7). * = Represents the mean of the blood glucose level and standard deviation of the mean in NOD mice from two weeks post diabetes onset till the end of the therapy is shown (week 21).
(DOCX)

Table S2 **The mouse immune response genes to AG490 treatment.** Female NOD mice were treated with AG490 or DMSO 3x/week for 5 consecutive weeks and then they were sacrificed one week after the last injection at week 10. Total RNA of purified CD4+ T-cells of splenocytes of treated mice and controls were pooled together and then subjected to real-time RT PCR using the mouse immune response genes array (Applied Biosystems) following manufacturer's instructions. Data analyzing was performed using integrated software based on expression of two housekeeping genes and then calibrated based on the control mice (DMSO treated mice). Shown represents fold change gene expression in AG490 treated NOD mice when compared with the sham treated mice.
(DOCX)
