# Supplementary material for: The Tyrphostin Agent AG490 Prevents and Reverses Type 1 Diabetes in NOD Mice
Source: PLoS One. 2012 May 14;7(5):e36079. doi: 10.1371/journal.pone.0036079 (PMC3351395; doi:10.1371/journal.pone.0036079)

**Figure S1- The effects of AG490 on members of the Jak-Stat in the pancreas.**

Prediabetic NOD mice (4 week) were treated either with AG490 or DMSO three times per week for 5 consecutive weeks and were sacrificed one week after the last injection at week 10. Pancreata were fixed and immunofluorescence staining was performed on microscopic slides as described in materials and methods. At least 30 islets per marker per group were analyzed.


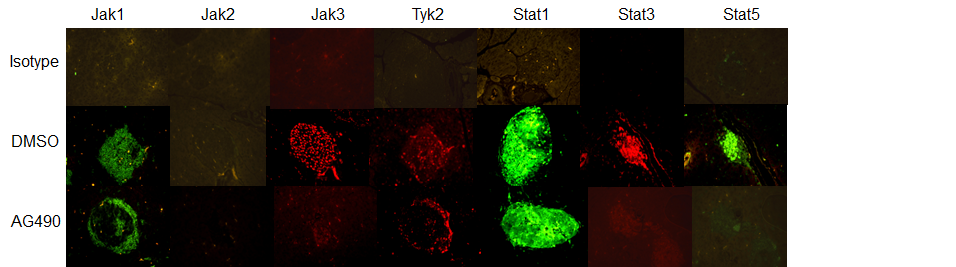

Supplement: Figure S1 — The effects of AG490 on members of the Jak-Stat in the pancreas. Prediabetic NOD mice (4 week) were treated either with AG490 or DMSO three times per week for 5 consecutive weeks and were sacrificed one week after the last injection at week 10. Pancreata were fixed and immunofluorescence staining was performed on microscopic slides as described in materials and methods. At least 30 islets per marker per group were analyzed. (DOCX) [file pone.0036079.s001.docx]
