# Supplementary material for: The Tyrphostin Agent AG490 Prevents and Reverses Type 1 Diabetes in NOD Mice
Source: PLoS One. 2012 May 14;7(5):e36079. doi: 10.1371/journal.pone.0036079 (PMC3351395; doi:10.1371/journal.pone.0036079)
Supplement: Table S1 — AG490 reverses diabetes in NOD mice. Reversal of diabetes was performed by administration of AG490 via i.p route. Recently diagnosed diabetic NOD mice (n = 23) were under AG490 treatment for a period of 21 weeks. The age of disease onset, blood glucose level at disease onset and the mean of blood glucose level after treatment and also total number of AG490 injections are shown. Bold cases represent NOD mouse in which AG490 was effective and successfully established euglycemia (n = 7). * = Represents the mean of the blood glucose level and standard deviation of the mean in NOD mice from two weeks post diabetes onset till the end of the therapy is shown (week 21). (DOCX) [file pone.0036079.s002.docx]

**Table S1- AG490 reverses diabetes in NOD mice-** Reversal of diabetes was performed by administration of AG490 via i.p route. Recently diagnosed diabetic NOD mice (n=23) were under AG490 treatment for a period of 21 weeks. The age of disease onset, blood glucose level at disease onset and the mean of blood glucose level after treatment and also total number of AG490 injections are shown. Bold cases represent NOD mouse in which AG490 was effective and successfully established euglycemia (n=7). * = Represents the mean of the blood glucose level and standard deviation of the mean in NOD mice from two weeks post diabetes onset till the end of the therapy is shown (week 21).

| **NO.** | **Age at diabetes onset**  **(wk)** | **# of AG490 injections** | **Blood glucose level at diabetes onset**  **(mg/dl)** | ***Blood glucose level post treatment**  **(mean±Std)** |
| --- | --- | --- | --- | --- |
| 1 | 20 | 13 | 351 | 597±4 |
| **2** | **20** | **26** | **306** | **188.45±67.54** |
| **3** | **21** | **17** | **319** | **141.50±32.46** |
| 4 | 20 | 13 | 406 | 599±0 |
| 5 | 24 | 13 | 376 | 593±12 |
| 6 | 20 | 14 | 268 | 555.5±87 |
| 7 | 30 | 15 | 375 | 595.2±8.5 |
| 8 | 22 | 6 | 378 | 599±0 |
| 9 | 16 | 12 | 367 | 592.8±13.86 |
| **10** | **32** | **5** | **311** | **140.68±39.94** |
| **11** | **33** | **8** | **277** | **182.2±70.38** |
| 12 | 25 | 10 | 290 | 521±72.38 |
| 13 | 30 | 18 | 296 | 556.33±61.23 |
| **14** | **35** | **7** | **318** | **173.22±42.78** |
| 15 | 20 | 12 | 309 | 538.5±121 |
| 16 | 21 | 10 | 356 | 585.2±28.67 |
| 17 | 34 | 10 | 335 | 571.25±55.50 |
| **18** | **35** | **5** | **374** | **192.875±82.15** |
| 19 | 25 | 16 | 277 | 369.6±157.64 |
| 20 | 40 | 8 | 478 | 563±0 |
| 21 | 30 | 5 | 423 | 599±0 |
| 22 | 41 | 8 | 344 | 429.67±46.76 |
| **23** | **48** | **2** | **298** | **152±9.87** |
