# Supplementary material for: The Tyrphostin Agent AG490 Prevents and Reverses Type 1 Diabetes in NOD Mice
Source: PLoS One. 2012 May 14;7(5):e36079. doi: 10.1371/journal.pone.0036079 (PMC3351395; doi:10.1371/journal.pone.0036079)
Supplement: Table S2 — The mouse immune response genes to AG490 treatment. Female NOD mice were treated with AG490 or DMSO 3x/week for 5 consecutive weeks and then they were sacrificed one week after the last injection at week 10. Total RNA of purified CD4+ T-cells of splenocytes of treated mice and controls were pooled together and then subjected to real-time RT PCR using the mouse immune response genes array (Applied Biosystems) following manufacturer's instructions. Data analyzing was performed using integrated software based on expression of two housekeeping genes and then calibrated based on the control mice (DMSO treated mice). Shown represents fold change gene expression in AG490 treated NOD mice when compared with the sham treated mice. (DOCX) [file pone.0036079.s003.docx]

**Table S2- The mouse immune response genes to AG490 treatment-** Female NOD mice were treated with AG490 or DMSO 3x/week for 5 consecutive weeks and then they were sacrificed one week after the last injection at week 10. Total RNA of purified CD4+ T-cells of splenocytes of treated mice and controls were pooled together and then subjected to real-time RT PCR using the mouse immune response genes array (Applied Biosystems) following manufacturer’s instructions. Data analyzing was performed using integrated software based on expression of two housekeeping genes and then calibrated based on the control mice (DMSO treated mice). Shown represents fold change gene expression in AG490 treated NOD mice when compared with the sham treated mice.

| **Name** | **Fold change** | **Std** | **Probe No.** |
| --- | --- | --- | --- |
| Ctla4 | 2.1465 | 0.054447 | Mm00486849_m1 |
| Cd3e | 1.331 | 0.033941 | Mm00599683_m1 |
| Gzmb | 1.2925 | 0.033234 | Mm00442834_m1 |
| Il12a | 1.188 | 0.029698 | Mm00434165_m1 |
| Cd4 | 1.18 | 0.029698 | Mm00442754_m1 |
| Fas | 1.1395 | 0.028991 | Mm00433237_m1 |
| Ccr4 | 1.1365 | 0.028991 | Mm00438271_m1 |
| H2-Eb1 | 1.12 | 0.028284 | Mm00439221_m1 |
| Cd28 | 1.1295 | 0.028991 | Mm00483137_m1 |
| Cd40lg | 1.1 | 0.028284 | Mm00441911_m1 |
| Cd86 | 1.065 | 0.02687 | Mm00444543_m1 |
| Csf1 | 1.0595 | 0.027577 | Mm00432688_m1 |
| Ccr7 | 1.0205 | 0.026163 | Mm00432608_m1 |
| Fasl | 0.9815 | 0.024749 | Mm00438864_m1 |
| Bax | 0.9675 | 0.024749 | Mm00432050_m1 |
| Icos | 0.896 | 0.022627 | Mm00497600_m1 |
| Ccr2 | 0.8935 | 0.023335 | Mm99999051_gH |
| Cd8a | 0.8595 | 0.02192 | Mm01182107_g1 |
| Cxcr3 | 0.8485 | 0.02192 | Mm00438259_m1 |
| Bcl2l1 | 0.82 | 0.021213 | Mm00437783_m1 |
| Cxcl10 | 0.774 | 0.019799 | Mm00445235_m1 |
| Bcl2 | 0.7335 | 0.019092 | Mm00477631_m1 |
| Ccl5 | 0.682 | 0.016971 | Mm01302428_m1 |
| Cd38 | 0.6395 | 0.016263 | Mm00483146_m1 |
| C3 | 0.6305 | 0.016263 | Mm00437858_m1 |
| Hmox1 | 0.6265 | 0.016263 | Mm00516004_m1 |
| Cd68 | 0.5725 | 0.014849 | Mm00839636_g1 |
| Cd19 | 0.56 | 0.014142 | Mm00515420_m1 |
